# Maternal Breast Growth and Body Mass Index Are Associated with Low Milk Production in Women

**DOI:** 10.3390/nu16172854

**Published:** 2024-08-26

**Authors:** Xuehua Jin, Ching Tat Lai, Sharon L. Perrella, Jacki L. McEachran, Zoya Gridneva, Donna T. Geddes

**Affiliations:** 1 School of Molecular Sciences, The University of Western Australia, Crawley, WA 6009, Australia; xuehua.jin@research.uwa.edu.au (X.J.); ching-tat.lai@uwa.edu.au (C.T.L.); sharon.perrella@uwa.edu.au (S.L.P.); jacki.mceachran@uwa.edu.au (J.L.M.); donna.geddes@uwa.edu.au (D.T.G.); 2 UWA Centre for Human Lactation Research and Translation, Crawley, WA 6009, Australia; 3 ABREAST Network, Perth, WA 6000, Australia

**Keywords:** lactation, human milk, milk production, body mass index, breast volume, breast growth

## Abstract

Background: Maternal breast volume is determined by the quantity of glandular and adipose tissue, and it undergoes significant changes during pregnancy. These changes are intricately linked to the development of glandular tissue, which most likely reflects lactation capacity. Evidence indicates that women with overweight or obesity exhibit larger breast volume compared to those with a normal body mass index (BMI), emphasizing the close relationship between breast volume and maternal adiposity. Hence, we aim to investigate breast volume growth and maternal BMI as potential risk factors for low milk production. Methods: Lactating women (n = 609) from the Perth metropolitan area in Western Australia between 2011 and 2023 were included in the analysis. Twenty-four-hour milk production measurements were conducted using the test weighing method, and milk removal frequencies were recorded. Mothers completed questionnaires regarding demographic, obstetric and infant details. Linear and logistic regression models were used to determine maternal and infant factors associated with milk production. Results: Here we show that increasing maternal age and BMI are associated with low milk production. Moreover, larger pre-pregnancy breast volume and breast growth are associated with both higher BMI and milk production. Conclusions: Women who are older, have an obese BMI and who have minimal pre-pregnancy breast volume and breast growth should be provided with antenatal screening and breastfeeding support as they are more likely to experience low milk production.

## 1. Introduction

Low milk production (LMP) poses a significant barrier to exclusive and continued breastfeeding [1]. From anatomical and physiological perspectives, sufficient breast development constitutes a fundamental prerequisite for optimal milk production (MP) capacity, while adequate and frequent milk removal is critical to full MP postpartum [2,3]. Under the influence of hormones, the main changes occurring in the mammary gland are initiated at puberty, including the formation of terminal end buds, ductal elongation and side branching. During pregnancy, changes in the breast, marked by rapid expansion of the ductular–lobular–alveolar system, lead to an increase in breast volume (BV) [4]. However, women with breast hypoplasia may exhibit impairment or absence of this developmental progress, leading to inadequate glandular tissue and, therefore, MP to meet their infants’ nutritional requirements [5].

The BV changes during pregnancy are related to glandular tissue development [6], which is a major determinant of lactation capacity. Several studies have shown that minimal BV growth (ΔBV) during pregnancy was associated with insufficient milk supply and a shorter duration of both exclusive and any breastfeeding [7,8]. Conversely, results from another study indicated that ΔBV during pregnancy was not related to the probability of establishing successful lactation [9]. Additionally, a study examining BV throughout lactation and comparing it to pre-pregnancy levels found no associations between increased BV with MP at any stage of lactation [10]. Up to now, there is no consensus in this regard, and more research needs to be carried out.

Adipose tissue contributes to BV, comprising up to 56% [11]. BV is affected by maternal adiposity, with evidence that women with overweight and obesity have 2–3 times greater BV than women with a normal body mass index (BMI) [12]. Interestingly, women with obesity are more likely to report limited breast growth during pregnancy [7]. This suggests the possibility of limited breast development, with reduced ductal branching and impaired alveolar development evident in rodent models [13], which show reduced lactation capacity in obese dams [14]. In women, obesity itself is widely recognized as a risk factor for suboptimal breastfeeding outcomes. Compared to women with a normal BMI, women within an overweight or obese BMI range are less likely to initiate, exclusively breastfeed or continue breastfeeding [15,16]. Furthermore, emerging evidence suggests that greater volumes of mammary adipose tissue in women with obesity may produce or store higher levels of metabolically active endogenous steroid hormones and environmental lipophilic chemicals, exerting an adverse impact on MP [17].

Considered together, BV and maternal adiposity are closely linked traits, with their interaction potentially impacting MP. The aim of this study was to investigate the relationships between MP, ΔBV from pre-pregnancy to postpartum and maternal postpartum BMI, potentially providing antenatally identifiable risk factors for LMP.

## 2. Materials and Methods

### 2.1. Participants

This is a retrospective cohort study involving lactating women whose 24 h MP and bra size data were collected between 2011 and 2023 in the Perth metropolitan area, Western Australia. Data were retrieved from studies that included women with no milk supply concerns as well as women with potential LMP. Exclusion criteria included preterm birth (birth gestation < 37 weeks), low infant birth weight (<2500 g), multiple births, smoking, history of nipple piercings and breast surgery. The final cohort included 609 participants. This study was conducted in accordance with the Declaration of Helsinki and was approved by the Human Research Ethics Committee at The University of Western Australia (2019/RA/4/20/6134). Informed written consent was obtained from all participants.

### 2.2. Data Collection

Given the stability in daily infant intake of human milk between 1–6 months postpartum [18], 24 h MP measurements were conducted within this temporal range using the test weighing method [19]. Briefly, pre- and post-feed weights of infants were recorded using electronic scales (±2.0 g; Electronic Baby Weigh Scale, Medela Inc., McHenry, IL, USA) for all feeds in one 24 ± 4 h period. Additionally, weights of milk collection bottles before and after any manual or pump breast expressions were also included in the assessment of *MP*. All removed milk weights were recorded in grams and expressed in milliliters based on a milk density of 1.03 g/mL [20]. The 24 h *MP* was calculated with the formula below, where *v_i_* is the volume of each feed/expression, *N* is the total number of feeds and expressions and *T* is the elapsed time from the end of the first feed until the end of the last feed.

$$MP=\sum_{i=2}^{N}v_{i}\frac{24}{T}$$

The cohort was classified into two groups: low milk production (LMP, <600 mL/24 h) and normal milk production (NMP, ≥600 mL/24 h). Milk removal frequency, including breastfeeding frequency and milk expression frequency, was recorded during the MP measurement process. Mothers completed questionnaires regarding demographic, obstetric and infant details, including maternal age, height, current weight, pre-pregnancy and postpartum bra size, parity, birth gestation, birth mode, infant birth weight and sex. Maternal BMI was calculated as kg/m^2^, and BV (cm^3^) of one breast was calculated based on both bra cup size and band size referring to the online chart (Table 1) [21]. ΔBV was calculated as postpartum BV minus pre-pregnancy BV.

### 2.3. Statistical Analyses

Descriptive statistics are presented as mean and standard deviation for continuous variables and frequencies and percentages for categorical variables. Correlation analysis was used to examine the interrelations among variables (Supplementary Material Figure S1). Univariable and multivariable regression analyses were used to assess the associations of maternal and infant characteristics with MP on both continuous (linear regression) and categorical (logistic regression) outcomes. All relevant covariates were initially included in the multivariable regression models. We used the Akaike Information Criterion (AIC) as a tool for the final model selection, opting for the model with the lowest AIC score [22]. Since AIC is sensitive to sample variability, to increase reliability, we repeated the selection procedure across 500 bootstrap resampling technique [23]. To be included in the final models, a covariate had to be selected by more than 50% of the bootstrapped models. The covariates selected from the bootstrapped models were the same as those identified by the lowest AIC. The Mann–Whitney U-test and post-hoc Bonferroni test were applied to elucidate differences between maternal BV and BMI groups. The significance level was set at *p* < 0.05, and all analyses were carried out in R Statistical Software 4.2.2 (R Foundation for Statistical Computing, Vienna, Austria).

## 3. Results

### 3.1. Descriptive Statistics

Characteristics of the mothers and infants are presented in Table 2 (n = 609). One hundred and seventy women (27.9%) were identified to have LMP and 439 women (72.1%) had NMP. Milk expression was recorded by 263 mothers (43.2%), including 24 (3.9%) who exclusively expressed. Of those with recorded BMI data, 205 women (42.1%) had a normal BMI (<25 kg/m^2^), while 282 (57.9%) were overweight or obese (≥25 kg/m^2^).

### 3.2. Maternal and Infant Factors Associated with Milk Production

Univariable linear regression analysis revealed significant negative associations of maternal age and BMI with MP (Table 3). Milk expression frequency was significantly positively associated with MP, with an average 5.5 mL/24 h produced with each additional expression frequency. Mothers of male infants had a significantly higher MP (50.5 mL/24 h on average) compared to mothers of female infants. After adjusting for potential confounders, the negative associations of maternal age and BMI with MP persisted. With every one-year increase in maternal age, there was an average 7.8 mL/24 h decrease in MP, while each one-unit increase in maternal BMI was associated with an 8.4 mL/24 h decrease in MP. Infant sex exhibited no significant association in the multivariable analysis. ΔBV showed small but significant positive associations with MP. Moreover, birth mode was significantly associated with MP, with MP after vaginal birth on average 45.1 mL/24 h lower than after C-section birth.

### 3.3. Maternal and Infant Factors Associated with the Likelihood of Low Milk Production

Table 4 presents the results of logistic regression analysis. In the univariable analysis, maternal age and BMI showed significant associations with LMP. Each one-year increase in age was associated with a 5.4% higher likelihood, and every 1 unit increase in BMI was associated with 7.3% higher likelihood of LMP. These associations remained significant after adjusting for potential confounders. However, breastfeeding frequency and parity, which exhibited significant associations with LMP in the univariable analysis, were not significant when other confounders were considered. Additionally, in the multivariable analysis, a 100 cm^3^ larger pre-pregnancy BV was associated with a 10% decrease in the odds of LMP, while a 100 cm^3^ larger ΔBV was associated with a 20% decrease in the likelihood of LMP.

### 3.4. Maternal Breast Volume Growth and Body Mass Index

**Figure 1:**
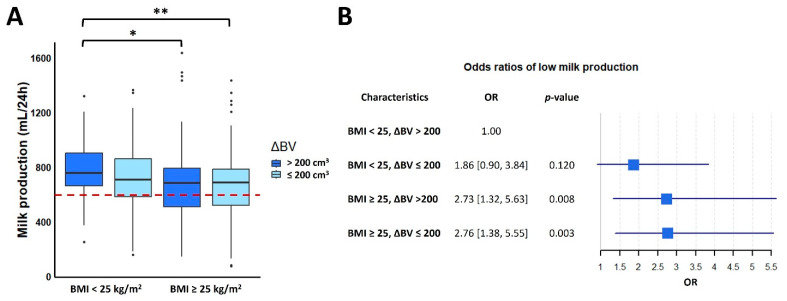
Maternal BMI and ΔBV in relation to milk production. (**A**) Interactive association of maternal BMI and ΔBV with MP. The box represents the interquartile range (IQR), with the line inside the box indicating the median. The whiskers extend to the lowest and highest data points within 1.5 times the IQR from the lower and upper quartiles, respectively. Data points beyond the whiskers are considered outliers. The red dashed line indicates 600 mL/24 h, which is the dividing line between low milk production and normal milk production. (**B**) Odds ratio of low milk production in different maternal BMI and ΔBV groups. BMI: body mass index; OR: odds ratio; ΔBV: breast volume growth; *: *p* < 0.05; **: *p* <0.01.

Women with an overweight or obese BMI had significantly higher BV at both pre-pregnancy and postpartum (*p* < 0.001), as well as higher ΔBV (*p* = 0.028). Although maternal BV typically increased from pre-pregnancy to postpartum, a small proportion of women (n = 83, 13.6%) reported no noticeable breast growth, while 47 women (7.7%) had ΔBV > 400 cm^3^. Interestingly, women who did not observe any breast growth exhibited the highest percentage of LMP (39.8%), and those with ΔBV > 400 cm^3^ had the highest percentage of NMP (80.9%). For women with ΔBV > 200 cm^3^ (Figure 1A), those with normal BMI had significantly higher MP than women with overweight or obese BMI (mean difference: 93.8 mL/24 h, *p* = 0.042). Both normal BMI and ΔBV > 200 cm^3^ had significantly higher MP compared to women with overweight or obese BMI and ΔBV ≤ 200 cm^3^ (mean difference: 108.1 mL/24 h, *p* = 0.006). Moreover, when compared with the group of normal BMI and ΔBV > 200 cm^3^, overweight or obese BMI groups were more than twice as likely to have LMP, even with adequate breast growth from pre-pregnancy to postpartum (Figure 1B).

## 4. Discussion

The findings of this study suggest that increased maternal age and BMI are associated with LMP. Furthermore, larger maternal pre-pregnancy BV and breast growth are associated with both higher BMI and MP. Overall, the study contributes valuable insights into the intricate relationships between maternal characteristics, reproductive BV dynamics and MP outcomes.

In our cohort, each one-year increase in maternal age is associated with a 5.4% higher likelihood of LMP. This aligns with the decline in MP observed with age in dairy cows [24]. Studies in women also suggest that increasing maternal age poses a risk for non-exclusive breastfeeding and shorter breastfeeding duration [25,26,27]. However, two small-scale studies measuring 24 h MP in women found no significant age-related associations [28,29]. Aging is accompanied by changes in body composition and a decline in basal metabolic rate [30], which might affect the overall energy balance available for lactation. Additionally, aging is associated with decreases in both insulin secretion and insulin sensitivity [31]. Lower insulin sensitivity may be linked to impaired secretory differentiation, with subsequent delayed secretory activation and reduced MP [32]. Furthermore, increasing maternal age is associated with significantly elevated risks for metabolic disorders and pregnancy complications, such as chronic/gestational hypertension, diabetes and excessive labor bleeding [33], all of which have been linked to impaired lactation [34]. It is important to note that whilst the association between maternal age and MP was statistically significant, the decrease in MP with each additional year of maternal age was only 7.8 mL/24 h. This represents 1.3% of 600 mL, which is not a substantial amount to worry about for women with NMP.

Our analysis revealed an unexpected finding that vaginal birth was associated with lower MP. Notably, no associations were found between vaginal birth and the likelihood of LMP in the logistic regression model. This suggests that birth mode is not a significant risk factor for LMP. Previous research has highlighted C-section as a potential variable hindering the onset of lactation [35,36]. Moreover, recent studies show that C-section is associated with lower rates of exclusive and any breastfeeding, as well as earlier cessation [36,37]. Our observation may be influenced by the possibility that women participating in our study were committed to breastfeeding, as indicated by their longer intended breastfeeding duration, with 73.8% planning to breastfeed for more than 12 months [38]. Additionally, 50.4% of women in our cohort had obtained a diploma, bachelor’s degree or higher education, likely increasing their awareness of the importance of frequent milk removal in the immediate postpartum period [39]. This underscores the need to intensively study breastfeeding behaviors in the first 2 weeks postpartum, which is critical to not only the initiation of lactation but the level of MP attained [40].

In agreement with previous reports [29,41], high BMI is an important risk factor for LMP. Insights from animal models reveal that obesity-induced lactogenesis impairment may result from altered mammary gland structure, such as fewer and smaller alveoli, larger adipose deposits and reduced ductal branching frequency [13,42]. Obesity is associated with diverse endocrine dysregulations. Elevated BMI is a recognized precursor to metabolic disorders, including gestational diabetes mellitus, type 2 diabetes mellitus and polycystic ovary syndrome, potentially driven by insulin deficiency or resistance [43,44]. Insulin metabolism plays a pivotal role in milk secretion, regulating genes involved in mammary epithelial cell proliferation and stimulating milk protein and lipid biosynthesis [45,46,47]. Moreover, reduced prolactin activity has been observed in lactating obese rodents and in women with obesity, which may be responsible for reduced lactogenesis and synthesis of milk components, as well as increased lipogenesis and lipid stored in adipocytes [48,49]. Recent evidence indicates that mammary adipose tissue in women with obesity may produce or store higher levels of estrogens, progesterone and external lipophilic chemicals, exerting an adverse impact on MP [17]. Additionally, women with overweight or obese BMI more frequently report postpartum body confidence issues and body image concerns [50,51], which are associated with both intended and actual shorter breastfeeding duration [52]. As presented in our study, women with higher BMI generally have larger breasts, and may find it more difficult to position their infants for breastfeeding [53,54]. Health care providers have noted that women with both obesity and large breasts face greater challenges in initiating breastfeeding compared to their counterparts with either obesity or large breasts alone [55]. This can be attributed to the combined effects of disrupted endocrine regulation induced by obesity and the psychological and practical challenges influencing autocrine control of milk synthesis.

While the significant changes in mammary gland development and breast size are initiated at puberty, during pregnancy, the breast attains its maximum development in response to hormonal stimulation [56]. Placental-derived estrogens and progesterone play a crucial role in stimulating extensive ductal growth, branching and lobuloalveolar development [57,58,59,60]. Recent evidence suggests that the pathways leading to either LMP or NMP start as early as puberty. It was reported that later menarche correlated with the absence of breast growth during both puberty and pregnancy, which further correlated with lower numbers of ducts with smaller diameters and, consecutively, with lower MP [61]. This partly explains our finding that lower pre-pregnancy BV was associated with LMP. Associations between breast growth during pregnancy and breastfeeding outcomes remain under debate. Some studies suggest no association between breast growth during pregnancy and 24 h MP or the probability of establishing successful lactation [9,62], while others propose that women with higher ΔBV may have a higher likelihood of successfully initiating and sustaining breastfeeding [7,8]. So far, limited studies have explored postpartum BV changes or engorgement in relation to lactation outcomes, including secretory activation and breastfeeding frequency. In our study, lower pre-pregnancy BV and ΔBV emerged as risk factors for LMP, albeit less significantly than the impact of high BMI. Given the strong relationship between BV, ΔBV and maternal BMI, the potential positive influence of higher BV or ΔBV on MP may be offset by the substantial adverse effects of obesity. Nevertheless, confirming normal breast glandular development during pregnancy is essential, as reflected in a higher incidence of LMP among women with no ΔBV. Furthermore, since more than 60% of women lacking breast growth successfully achieved NMP, it’s important to note that the absence of ΔBV doesn’t necessarily indicate underdeveloped mammary glands. Some women with no ΔBV during pregnancy may still notice breast changes such as increased density and heaviness. These changes signify the development of glandular tissue and the potential for NMP. However, mammary hypoplasia, an uncommon condition with an uncertain etiology, can impede breast growth during pregnancy and result in insufficient glandular tissue for NMP [63,64]. Features of breast hypoplasia may include a wide intermammary space, breast asymmetry and a tubular shape of the breasts [5]. Clinically, the development of breast glandular tissue can be assessed through inspection, palpation and various imaging techniques [65,66]. Women who lack breast growth during pregnancy and suspect breast hypoplasia should seek evaluation of their glandular development and guidance from maternity care providers.

A notable strength of our study is the substantial sample size, which enhances the validity and precision of our study results, contributing to a more thorough understanding of the relationships between BV and MP. Another strength lies in our approach to measuring actual 24 h MP using a validated reference method [67]. Due to the practical difficulties of 24 h MP measurements, such as high cost and participant compliance, the reported outcomes of lactation-related studies are usually limited to durations of exclusive or any breastfeeding. To our knowledge, only one study prior to ours has measured 24 h MP during established lactation and explored its relationship with ΔBV during pregnancy [62]. However, this study was limited by a small sample size of only 8 participants, thereby constraining the statistical power of their findings.

We acknowledge a limitation in the reliance on self-reported bra size, height and weight, which may introduce potential biases [68]. Factors such as wearing the wrong-sized bra [69] or an old bra with a loose band could contribute to inaccuracies in the recorded data. Additionally, collecting pre-pregnancy bra size data postpartum may have led to recall bias. Moreover, BV was a crude calculation, and there are likely some design differences between bra manufacturers. However, we believe that this approach allows women to estimate BV without any technical assistance, and an accurate measurement of breast volume is difficult to achieve [10]. Future studies might consider implementing more standardized measurement procedures such as three-dimensional scanning or computerized measurement to mitigate this limitation. Furthermore, investigating changes in breast density and heaviness as well as BV could provide valuable insights that assist in distinguishing between cases of no actual breast growth but perceived glandular development and cases where neither growth nor development have occurred in pregnancy. Lastly, the 600 mL threshold used to define LMP and NMP might not capture all individual variations, as 600 mL may be insufficient for optimal growth in some infants. Including infant growth data in future research could further refine the differentiation between LMP and NMP.

## 5. Conclusions

Women who are older, have an obese BMI and who have minimal pre-pregnancy BV and breast growth have a higher risk of LMP, and so antenatal screening may assist in timely detection and interventions to ensure the mutual health benefits of mothers and infants.

## Figures and Tables

**Table 1:** Breast volume corresponding to bra size.

Breast Volume of One Breast (cm^3^)	Australian Bra Size	US Bra Size
180	8A, 10AA	30A, 32AA
240	8B, 10A, 12AA	30B, 32A, 34AA
310	8C, 10B, 12A	30C, 32B, 34A
390	8D, 10C, 12B, 14A	30D, 32C, 34B, 36A
480	8DD/E, 10D, 12C, 14B, 16A	30E, 32D, 34C, 36B, 38A
590	8F, 10DD/E, 12D, 14C, 16B, 18A	30F, 32E, 34D, 36C, 38B, 40A
710	10F, 12DD/E, 14D, 16C, 18B	32F, 34E, 36D, 38C, 40B
850	10G, 12F, 14DD/E, 16D, 18C, 20B	32G, 34F, 36E, 38D, 40C, 42B
1000	10H, 12G, 14F, 16DD/E, 18D, 20C	32H, 34G, 36F, 38E, 40D, 42C
1180	12H, 14G, 16F, 18DD/E, 20D	34H, 36G, 38F, 40E, 42D
1370	12I, 14H, 16G, 18F, 20DD/E	34I, 36H, 38G, 40F, 42E
1580	14I, 16H, 18G, 20F, 22DD/E	36I, 38H, 40G, 42F, 44E
1810	14J, 16I, 18H, 20G, 22F	36J, 38I, 40H, 42G, 44F
2060	16J, 18I, 20H, 22G	38J, 40I, 42H, 44G

**Table 2:** Maternal and infant characteristics.

Characteristics	Mean ± SD (Range, n) or Number (%)
Birth gestation (weeks)	39.0 ± 1.1 (37–43, 609)
Infant age at MP measurement (months)	2.9 ± 1.2 (1–6, 609)
MP (mL/24 h)	718 ± 237 (48–1682, 609)
Milk removal frequency (times/24 h)	13.4 ± 4.9 (2–40, 609)
Breastfeeding frequency (times/24 h)	11.1 ± 4.6 (0–32, 609)
Milk expression frequency (times/24 h)	2.3 ± 3.8 (0–20, 609)
Maternal age (years)	33.3 ± 4.4 (20.6–48.2, 605)
Maternal BMI (kg/m^2^)	27.1 ± 5.6 (16.9–64.5, 487)
Pre-pregnancy BV (cm^3^)	554 ± 244 (180–2060, 609)
Postpartum BV (cm^3^)	745 ± 274 (240–2060, 609)
ΔBV (cm^3^)	192 ± 136 (−230–730, 609)
Parity: primiparous	373 (61.3%)
Birth mode: vaginal	380 (63.8%)
Infant sex: male	312 (51.4%)
Infant birth weight (g)	3443 ± 449 (2510–5045, 598)

BMI: body mass index; BV: breast volume; MP: milk production.

**Table 3:** Associations between maternal and infant characteristics and milk production.

Characteristics	Univariable Analysis			Multivariable Analysis		
	Coefficient	95% CI	*p*-Value ^†^	Coefficient	95% CI	*p*-Value ^†^
Maternal age (years)	−6.977	−11.221, −2.734	**0.001**	−7.770	−12.519, −3.020	**0.001**
Maternal BMI (kg/m^2^)	−5.321	−9.042, −1.601	**0.005**	−8.416	−13.613, −3.218	**0.002**
Pre-pregnancy BV (cm^3^)	−0.064	−0.141, 0.014	0.107	0.100	−0.015, 0.216	0.088
Postpartum BV (cm^3^)	−0.022	−0.091, 0.047	0.531	-	-	-
ΔBV (cm^3^)	0.115	−0.023, 0.253	0.104	0.168	0.019, 0.316	**0.027**
Breastfeeding frequency (times/24 h)	−1.702	−5.827, 2.423	0.418	-	-	-
Milk expression frequency (times/24 h)	5.508	0.525, 10.490	**0.030**	-	-	-
Milk removal frequency (times/24 h)	1.803	−2.067, 5.673	0.361	-	-	-
Infant birth weight (g)	0.014	−0.027, 0.056	0.497	-	-	-
Infant sex (Female vs. Male)	−50.490	−88.066, −12.918	**0.009**	−35.067	−76.048, 5.914	0.093
Birth mode (Vaginal vs. C-section)	−22.420	−61.645, 16.801	0.262	−45.108	−88.499, −1.717	**0.042**
Parity (Primiparous vs. Multiparous)	19.960	−18.788, 58.703	0.312	-	-	-

-: not included in the multivariable model; ^†^: bold font indicates statistically significant difference; BMI: body mass index; BV: breast volume; CI: confidence interval.

**Table 4:** Association between maternal and infant characteristics and the likelihood of low milk production.

Characteristics	Univariable Analysis			Multivariable Analysis		
	OR	95% CI	*p*-Value ^†^	OR	95% CI	*p*-Value ^†^
Maternal age (years)	1.054	1.013, 1.097	**0.010**	1.056	1.007, 1.107	**0.024**
Maternal BMI (kg/m^2^)	1.073	1.035, 1.111	**<0.001**	1.117	1.060, 1.177	**<0.001**
Pre-pregnancy BV (cm^3^)	1.001	1.000, 1.001	0.145	0.999	0.997, 1.000	**0.014**
Postpartum BV (cm^3^)	1.000	1.000, 1.001	0.733	-	-	-
ΔBV (cm^3^)	0.999	0.997, 1.000	0.054	0.998	0.997, 1.000	**0.036**
Breastfeeding frequency (times/24 h)	0.955	0.918, 0.994	**0.023**	0.958	0.917, 1.001	0.058
Milk expression frequency (times/24 h)	1.042	0.995, 1.088	0.081	-	-	-
Milk removal frequency (times/24 h)	0.985	0.949, 1.023	0.435	-	-	-
Infant birth weight (g)	1.000	1.000, 1.000	0.975	-	-	-
Infant sex (Female vs. male)	1.063	0.745, 1.516	0.735	-	-	-
Birth mode (Vaginal vs. C-section)	0.895	0.619, 1.294	0.555	-	-	-
Parity (Primiparous vs. Multiparous)	0.682	0.476, 0.976	**0.037**	0.731	0.479, 1.117	0.147

-: not included in the multivariable model; ^†^: bold font indicates statistically significant difference; BMI: body mass index; BV: breast volume; CI: confidence interval; OR: odds ratio.

## Data Availability

The datasets used and/or analysed during the current study are available from the corresponding author on reasonable request.

## Supplementary Materials

The following supporting information can be downloaded at: https://www.mdpi.com/article/10.3390/nu16172854/s1, Figure S1: Correlation analysis of milk production and maternal and infant characteristics.
