# Supplementary material for: Maternal Breast Growth and Body Mass Index Are Associated with Low Milk Production in Women
Source: Nutrients. 2024 Aug 26;16(17):2854. doi: 10.3390/nu16172854 (PMC11397153; doi:10.3390/nu16172854)
Supplement: Supplementary file 1 [file nutrients-16-02854-s001.zip › nutrients-3169574-supplementary.pdf]

Maternal Breast Growth and Body Mass Index Are Associated with Low Milk Production in Women

Xuehua Jin, Ching Tat Lai, Sharon L. Perrella, Jacki L. McEachran, Zoya Gridneva and Donna T. Geddes

Supplementary Figure S1. Correlation analysis of milk production and maternal and infant characteristics.

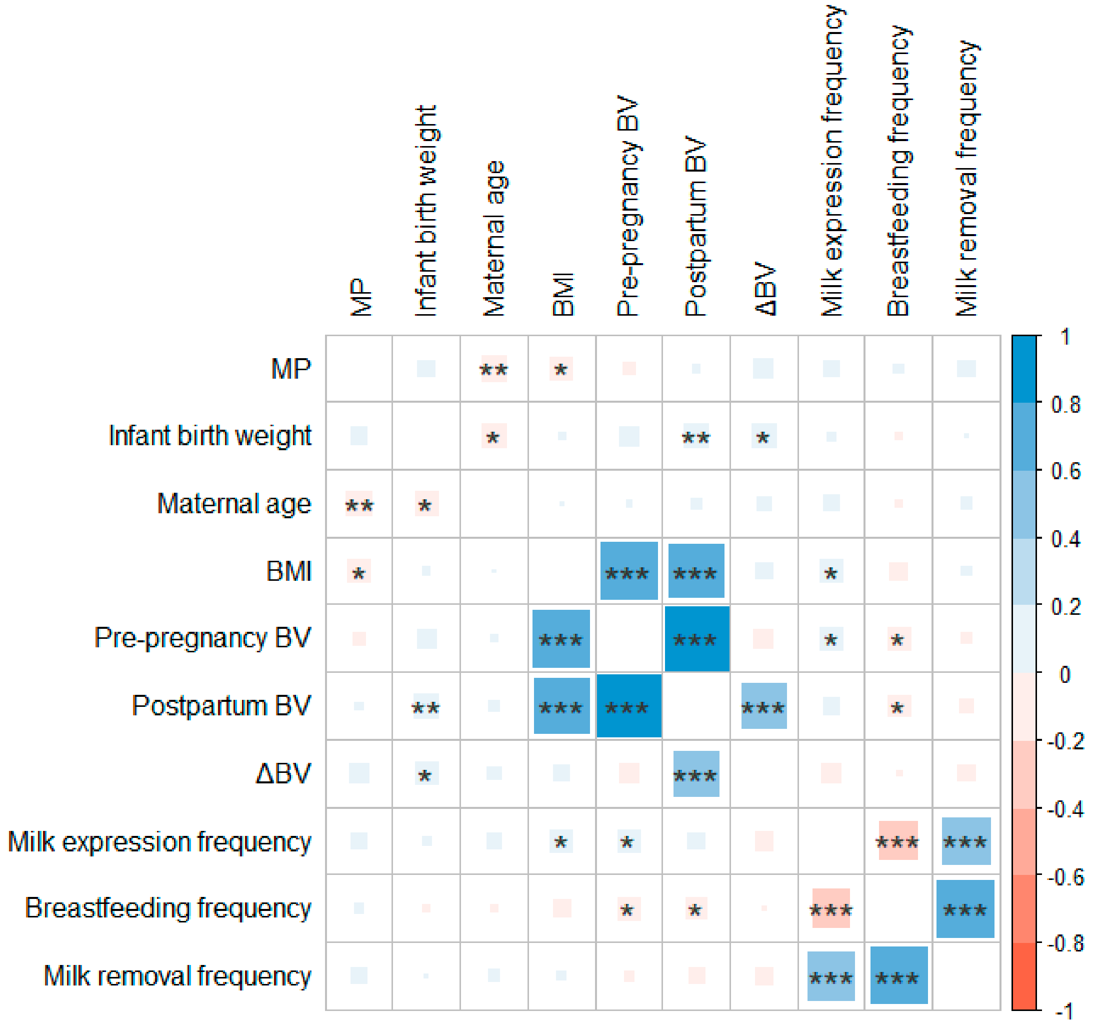

Figure S1. Correlation analysis of milk production and maternal and infant characteristics. Positive correlations are indicated in blue, and negative correlations in red. Darker and larger shades represent stronger correlations. BMI: body mass index; BV: breast volume; MP: milk production. * $p < 0.05$, ** $p < 0.01$, *** $p < 0.001$.
